# Longitudinal Changes in a Claims-Based Frailty Proxy Measure Compared to Concurrent Changes in the Fried Frailty Phenotype

**DOI:** 10.1093/gerona/glae174

**Published:** 2024-07-12

**Authors:** Emilie D Duchesneau, Dae Hyun Kim, Til Stürmer, Katherine Reeder-Hayes, Jessie K Edwards, Keturah R Faurot, Jennifer L Lund

**Affiliations:** Department of Epidemiology and Prevention, Division of Public Health Sciences, Wake Forest University School of Medicine, Winston-Salem, North Carolina, USA; Marcus Institute for Aging Research, Hebrew SeniorLife, Harvard Medical School, Roslindale, Massachusetts, USA; Division of Gerontology, Department of Medicine, Beth Israel Deaconess Medical Center, Brookline, Massachusetts, USA; Lineberger Comprehensive Cancer Center, University of North Carolina at Chapel Hill, Chapel Hill, North Carolina, USA; Department of Epidemiology, Gillings School of Global Public Health, University of North Carolina at Chapel Hill, Chapel Hill, North Carolina, USA; Lineberger Comprehensive Cancer Center, University of North Carolina at Chapel Hill, Chapel Hill, North Carolina, USA; Division of Oncology, Department of Medicine, University of North Carolina at Chapel Hill, Chapel Hill, North Carolina, USA; Department of Epidemiology, Gillings School of Global Public Health, University of North Carolina at Chapel Hill, Chapel Hill, North Carolina, USA; Department of Physical Medicine and Rehabilitation, School of Medicine, University of North Carolina at Chapel Hill, Chapel Hill, North Carolina, USA; Lineberger Comprehensive Cancer Center, University of North Carolina at Chapel Hill, Chapel Hill, North Carolina, USA; Department of Epidemiology, Gillings School of Global Public Health, University of North Carolina at Chapel Hill, Chapel Hill, North Carolina, USA

**Keywords:** Claims data, Longitudinal study, Medicare

## Abstract

**Background:**

Frailty is a dynamic aging-related syndrome, but measuring frailty transitions is challenging. The Faurot frailty index is a validated Medicare claims-based frailty proxy based on demographic and billing information. We evaluated whether 3-year changes in the Faurot frailty index were consistent with concurrent changes in the frailty phenotype in a cohort of older adults.

**Methods:**

We used longitudinal data from the National Health and Aging Trends Study (NHATS) with Medicare claims linkage (2010–2018). We identified older adults (66+ years) in the 2011 and 2015 NHATS cohorts with at least 1 year of Medicare fee-for-service continuous enrollment (*N* = 6 951). We described annual changes in mean claims-based frailty for up to 3 years, based on concurrent transitions in the frailty phenotype.

**Results:**

At baseline, 32% were robust, 48% prefrail, and 19% frail based on the frailty phenotype. Mean claims-based frailty for older adults who were robust at baseline and worsened to frail increased over 3 years (0.09–0.25). Similarly, those who worsened from prefrail to frail experienced an increase in mean claims-based frailty (0.14–0.26). Improvements in the frailty phenotype did not correspond to decreases in claims-based frailty. Older adults whose frailty phenotype improved over time had a lower baseline claims-based frailty score than those who experienced stable or worsening frailty.

**Conclusions:**

Older adults who experienced a frailty phenotype worsening over 3 years experienced concurrent increases in the Faurot frailty index. Our results suggest that claims data may be used to identify clinically meaningful worsening in frailty.

Frailty, a state of reduced physiologic reserve to maintain homeostasis, is a key manifestation of the biological aging process (1). The gold standard for measuring frailty is the Fried frailty phenotype, which categorizes individuals as robust, prefrail, or frail based on 5 clinical symptoms (1).

Frailty is dynamic and improvements and worsening in frailty are common in older adults (2). Identifying interventions to prevent frailty progression may help reduce adverse health outcomes and healthcare spending. However, measuring longitudinal frailty transitions is difficult, because clinical tools, such as the frailty phenotype, are time-consuming and expensive to administer (3). Medicare claims and enrollment data may offer a solution for studying changes in frailty over time.

The Faurot frailty index is a validated Medicare claims-based proxy measure (4–6). It has been used extensively in epidemiologic research to identify frail populations or to control for confounding by frailty (7–9). Its suitability for identifying clinically meaningful longitudinal changes in frailty has not been assessed. In this study, we evaluated whether 3-year changes in the Faurot frailty index were consistent with concurrent changes in the frailty phenotype in older Medicare beneficiaries.

## Method

### Data Source

We leveraged data from Rounds 1–8 (2011–2018) of the National Health and Aging Trends Study (NHATS) with linkage to Medicare claims and enrollment data (2010–2018). NHATS is a prospective, longitudinal cohort that conducts annual interviews for a sample of Medicare beneficiaries (10,11). The initial cohort was enrolled in 2011 and the cohort was replenished in 2015. We used linkage between NHATS and Medicare data for the subsample of NHATS participants enrolled in fee-for-service plans.

### Population and Study Design

**Figure 1.**
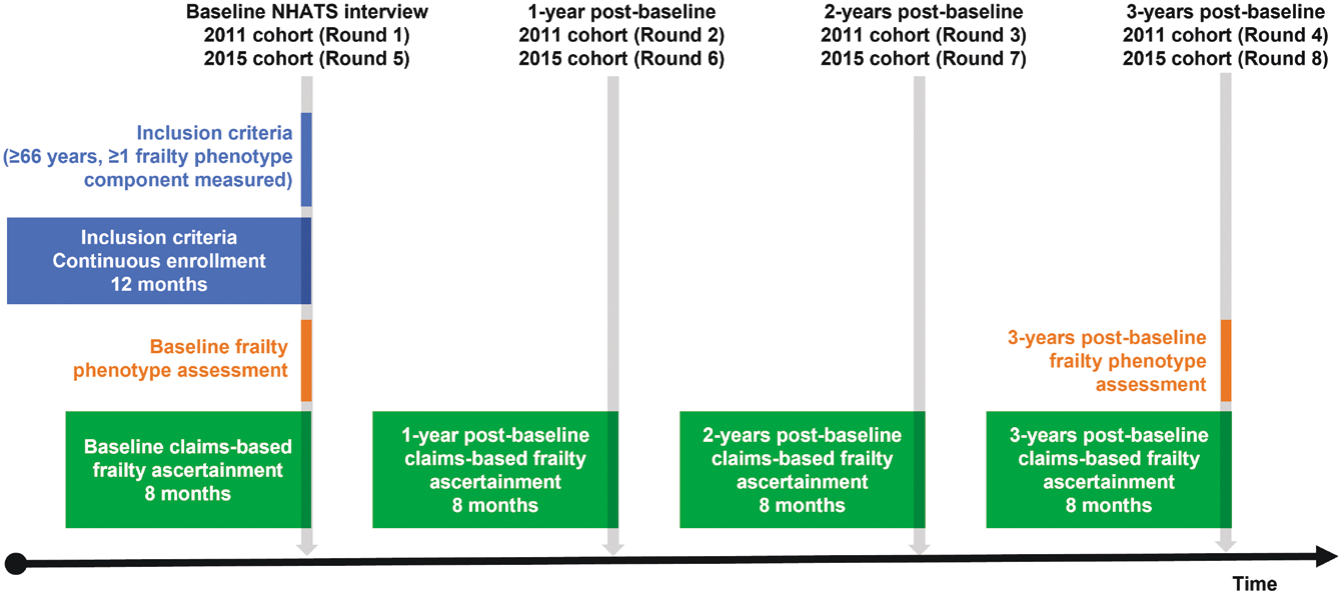
Study schematic. NHATS = National Health and Aging Trends Study.

We included participants in the 2011 and 2015 NHATS cohorts who were aged 66 years or older and resided in the community or a non-nursing home residential care facility at the time of the baseline interview. We required continuous enrollment in Medicare fee-for-service for at least 12 months prior to the baseline interview (2011 cohort: Round 1; 2015 cohort: Round 5). Individuals in the 2011 cohort who remained in NHATS during Round 5 were only included once. Participants were followed for up to 3 years or until death, loss to follow-up from NHATS, or disenrollment from Medicare fee-for-service (Figure 1) (12).

### Fried Frailty Phenotype

The frailty phenotype was the gold standard frailty measure (1). The 5 components of the frailty phenotype (exhaustion, low physical activity, shrinking, slowness, weakness) are assessed annually in NHATS using self-report and physical performance-based measures (13). The frailty phenotype was categorized as robust (0 frailty components), prefrail (1–2), and frail (3–5) (1). The frailty phenotype was assessed during the baseline interview and 3-years post-baseline (2011 cohort: Round 4; 2015 cohort: Round 8). Individuals who did not complete grip strength or walking speed tests due to physical inability were considered to meet the definitions for weakness and slowness, respectively (14). We imputed other missing frailty phenotype components probabilistically using hot-deck imputation, a method that has been previously described in NHATS (15). Individuals with missing frailty components were assigned the frailty phenotype of a randomly matched individual who shared the same pattern for nonmissing frailty components but had fully observed information.

### Claims-Based Frailty Measure

Claims-based frailty was assessed using the Faurot frailty index, a validated Medicare claims-based frailty measure that parametrically calculates a predicted probability of frailty using demographic information and diagnosis, procedure, and durable medical equipment codes (4–6). The original model was developed and validated as a predictor of dependences in the activities of daily living as a proxy for frailty (4). It has been externally validated as a predictor of the frailty phenotype in the Atherosclerosis Risk in Communities cohort (*C*-statistic: 0.71) (5) and in the NHATS–Medicare cohort (*C*-statistic: 0.75) (16).

The predicted probability of frailty is a continuous measure (range: 0–1), with a higher score indicating a higher likelihood of frailty. Prior studies have used a cut point of 0.20 to define a high predicted probability of frailty, as those with a score ≥0.20 are more likely to be frail, have dependencies in the activities of daily living, and have higher risks of falls, hospitalizations, and skilled nursing facility (SNF) admissions than those with lower predicted probabilities of frailty (4–6). Additional details about the development and validation of the Faurot frailty index are provided in the Supplementary Material. We calculated claims-based frailty on the date of each NHATS interview (baseline and annually for up to 3 years) using claims during the 240 days prior to the date of the corresponding interview.

### Covariates

Age, gender, self-reported race and ethnicity, region, residential setting, possible and probable dementia (17), history of fractures and falls, and mobility devices were assessed using the baseline NHATS survey. Time-varying comorbidities were assessed using the Gagne combined comorbidity score (18,19) and healthcare utilization was assessed based on the number of inpatient, outpatient, and emergency department visits during the 365 days prior to each NHATS interview.

### Statistical Analysis

Analyses were conducted using SAS version 9.4 (SAS Institute, Cary, NC). This study was approved by the University of North Carolina Institutional Review Board (IRB #21-0520).

We used inverse probability of attrition weighting (IPAW) to account for potentially informative loss to follow-up in NHATS, disenrollment from Medicare fee-for-service, and death during the follow-up period (20). IPAW upweights individuals who remain in a study to stand in for similar individuals who die or are lost to follow-up. We estimated the weights by fitting separate pooled logistic models for each type of attrition with the covariates as predictors, stratifying by baseline frailty phenotype.

As the reference standard, we used transitions in the frailty phenotype using a 9-item categorical variable based on the starting and ending phenotype categories over the 3-year period (eg, robust → prefrail, frail → frail). We estimated the mean claims-based frailty score for each of the 9 longitudinal frailty phenotype categories during each year of follow-up to evaluate whether we observed similar patterns as compared with the reference standard. The mean change in claims-based frailty over 3 years and corresponding 95% confidence intervals (CIs) were estimated using generalized estimating equations with a gamma distribution and identity link and a within-subject autoregressive correlation structure, which accounted for repeated observations and the skewed nature of the frailty score.

### Sensitivity Analysis

We conducted a sensitivity analysis with death considered as a separate health state. Claims-based frailty for individuals at the time of death was calculated using the claims during the 240 days prior to and including the date of death. This value was carried forward for all follow-up years following death. Additional details are provided in Supplementary Figure 1.

## Results

### Study Population

Our sample included 6 951 older adults (68% in the 2011 cohort, 32% in the 2015 replenishment cohort; Supplementary Figure 2). Table 1 presents baseline characteristics of the study population. The median age was 77 years (interquartile range: 71–84) and 58% were female. At baseline, 931 (15%) of older adults were missing at least 1 frailty phenotype component (weakness: 9%, slowness; 8%, shrinking: 3%, exhaustion: <1%, low physical activity: <1%). After hot-deck imputation, 32%, 49%, and 19% of older adults were robust, prefrail, and frail, respectively. The mean claims-based frailty score at baseline was 0.10 (median: 0.04) and differed by baseline frailty phenotype (mean robust: 0.04; prefrail: 0.09; and frail 0.24).

**Table 1.** Baseline Characteristics of Older Adults in the National Health and Aging Trends Study (2011 and 2015 Cohorts), *N* = 6 951

Characteristic^a^	*N* (%)
NHATS cohort	
2011	4 739 (68.2)
2015	2 212 (31.8)
Age, median (IQR)	77 (71, 84)
Gender	
Female	4 025 (57.9)
Male	2 926 (42.1)
Hispanic ethnicity	306 (4.5)
Self-reported race	
White	5 177 (76.0)
Black	1 359 (19.9)
Asian or Pacific Islander	123 (1.8)
American Indian or Alaska Native	66 (1.0)
Other	89 (1.3)
Place of residence	
Community	6 537 (94.0)
Non-nursing home residential care	414 (6.0)
Gagne combined comorbidity score	
≤0	3 249 (46.7)
1	1 113 (16.0)
2	718 (10.3)
≥3	1 871 (26.9)
NHATS dementia classification	
Probable dementia	830 (12.2)
Possible dementia	901 (13.2)
No dementia	5 087 (74.6)
Self-reported history of fractures and falls	
History of hip fracture since age 50	379 (5.5)
Fall during past month	786 (11.3)
Fall during past year	2 292 (33.0)
Mobility or walking devices in prior month	
Any mobility device	2 196 (31.6)
Cane	1 494 (21.5)
Walker	1 101 (15.8)
Wheelchair	590 (8.5)
Scooter	183 (2.6)
Healthcare resource utilization in prior year	
Any outpatient visit	6 416 (92.3)
Number of outpatient visits, median (IQR)	7 (3, 12)
Any ED visit	2 227 (32.0)
Any hospital admission	1 303 (18.7)
Fried frailty phenotype^b^	
Robust	2 198 (31.6)
Prefrail	3 401 (48.9)
Frail	1 352 (19.5)

*Notes*: ED = emergency department; IQR = interquartile range; NHATS = National Health and Aging Trends Study.

^a^Numbers may not sum to the total population size due to missing data.

^b^931 (15%) of older adults were missing at least 1 frailty phenotype component. The numbers and percentages in the table reflect the distribution after hot-deck imputation.

### Frailty State Transitions

Attrition was common in the 3-year period; 5%, 13%, and 32% of robust, prefrail, and frail older adults died during the 3-year follow-up period; 32%, 32%, and 29% were lost to follow-up from NHATS; and 7%, 6%, and 5% disenrolled from Medicare fee-for-service coverage.

**Figure 2.**
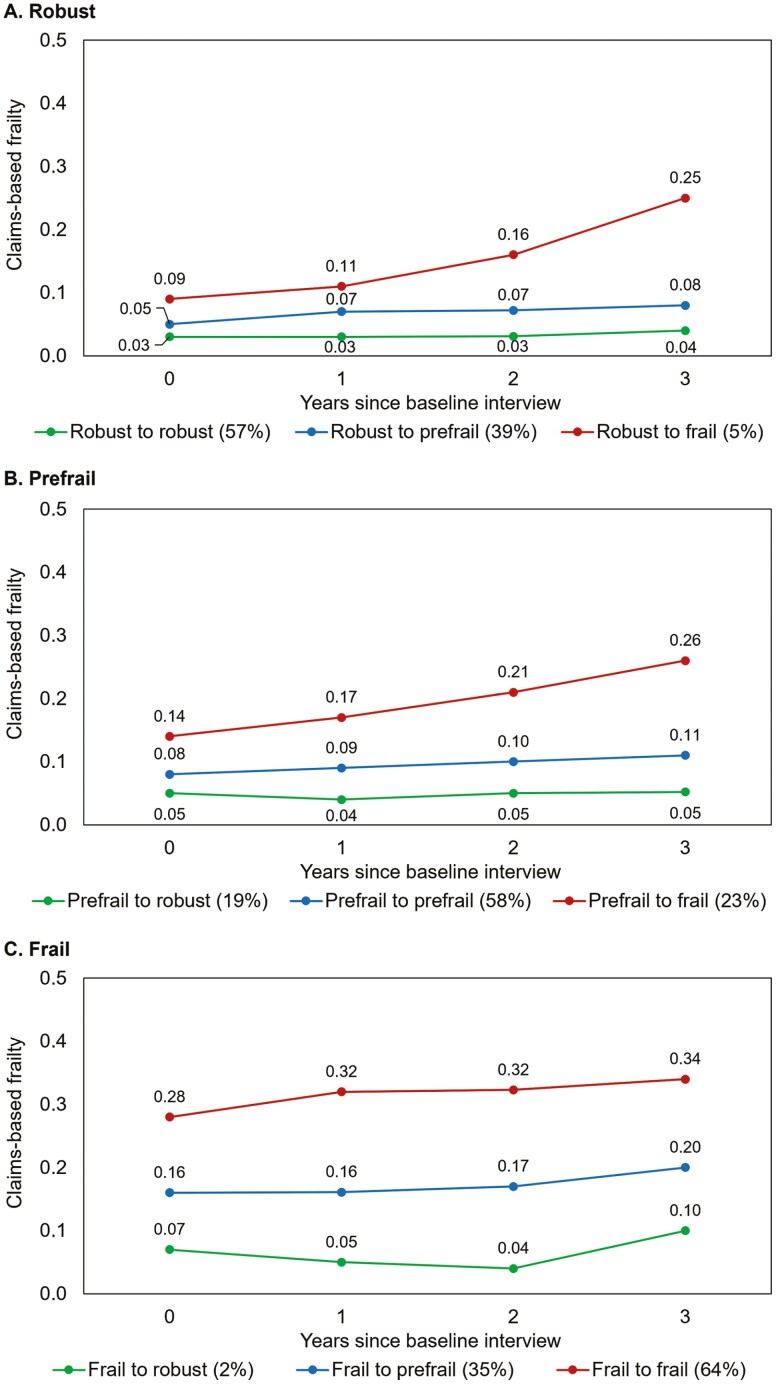
Three-year changes in claims-based frailty, stratified by transitions in the gold standard frailty phenotype.

After applying the attrition weights, 57% of robust individuals stayed robust after 3 years, 39% worsened to prefrail, and 5% worsened to frail (Figure 2, Panel A). Older adults who stayed robust had stable claims-based frailty over the 3-year follow-up (0.03–0.04; change = 0.01, 95% CI: 0.00 to 0.01). Older adults who worsened to prefrail experienced increases in mean claims-based frailty (0.05–0.08; change = 0.03, 95% CI: 0.02 to 0.04). Similarly, older adults who worsened to frail over 3 years experienced a larger increase in claims-based frailty (0.09–0.25; change = 0.15, 95% CI: 0.09 to 0.22).

Among prefrail individuals at baseline, 58% stayed prefrail, 19% improved to robust, and 23% worsened to frail (Figure 2, Panel B). Older adults who improved from prefrail to robust had stable claims-based frailty (0.05–0.05; change = 0.00, 95% CI: −0.00 to 0.01). Those who stayed prefrail had a slight increase in mean claims-based frailty (0.08–0.11; change = 0.03, 95% CI: 0.02 to 0.04). Those who worsened to frail had a larger increase in mean claims-based frailty (0.14–0.26; change = 0.12, 95% CI: 0.09 to 0.15).

Among frail individuals at baseline, 64% stayed frail, 2% improved to robust, and 35% improved to prefrail (Figure 2, Panel C). Older adults who improved from frail at baseline to robust experienced a slight increase in mean claims-based frailty (0.07–0.10; change = 0.03, 95% CI: −0.06 to 0.13). Similarly, older adults who improved from frail to prefrail also experienced a slight increase in claims-based frailty (0.16–0.20; change = 0.04, 95% CI: 0.00 to 0.08), although the results were imprecise. Older adults who remained frail experienced a larger increase in claims-based frailty over the 3-year period (0.28–0.34; change = 0.06, 95% CI: 0.03 to 0.09).

In the sensitivity analysis considering death as its own state, individuals who died during follow-up had higher mean claims-based frailty that increased more rapidly than those who remained alive at all time points (Supplementary Figure 3). Changes in mean claims-based frailty among older adults who remained alive were similar as in the primary analysis using IPAW.

## Discussion

We described patterns of change in a claims-based frailty measure relative to concurrent changes in a gold standard frailty measure in a large cohort of older adults. Older adults who worsened from robust or prefrail to frail during follow-up experienced substantial increases in their claims-based frailty score, suggesting that the claims-based measure can be used to identify clinically meaningful worsening in frailty. Individuals who remained frail throughout follow-up also experienced an increase in claims-based frailty, which may reflect further progression of frailty over time in these older adults.

Improvements in the frailty phenotype (eg, frail to prefrail) did not correspond to improvements in claims-based frailty. This finding may partially be explained by age being included as an indicator in the Faurot frailty algorithm. In addition, diagnosis codes from past healthcare encounters may be carried forward in medical charts and included in future claims, despite improvements in health status. Future work may consider refining claims-based frailty indices to better capture frailty improvements by including additional dimensions such as setting, timing, and frequency of claims.

Baseline claims-based frailty scores were notably higher in older adults who experienced a frailty worsening than those who had stable or improved frailty. For example, prefrail individuals who transitioned to the frail state had an average baseline claims-based frailty score of 0.14, compared to those who improved to robust (0.05) or stayed prefrail (0.08). This suggests that the baseline claims-based frailty scores may identify important heterogeneity within the gold standard phenotypic categories.

Attrition due to death, loss to follow-up, and disenrollment from Medicare fee-for-service were common during the 3-year follow-up period. Prior research has shown that in the NHATS cohort, the reasons why older adults are lost to follow-up differ by frailty status, with frail older adults being more likely to be lost to follow-up due to medical conditions (15). Although we tried to address this using IPAW, it is possible that we did not fully capture the variables related to attrition and residual bias may remain. Because individuals who die may be inherently different than those who remain alive, we also conducted a sensitivity analysis that treated death as its own health state and used the 240 days prior to death to calculate the final claims-based frailty score for these individuals. Unsurprisingly, older adults who died had higher claims-based frailty scores than those who remained alive and experienced larger increases in claims-based frailty up until their deaths.

We chose to evaluate 3-year changes in the frailty phenotype to identify long-term health trajectories and to allow sufficient opportunities for transitions in the frailty phenotype. This longer period also allowed sufficient time for the claims-based frailty index to reflect the true changes in frailty, because the claims-based measure requires a retrospective frailty ascertainment window. Future work using transition models, such as Markov models, may provide additional insight into whether the Faurot frailty index can be used to model shorter-term changes in the frailty phenotype. We described changes in the frailty phenotype based on changes between the baseline and 3-year measures, rather than assessing more granular changes. For example, the following frailty phenotype transitions in NHATS were included in the same category in our analysis: Robust → Prefrail → Frail → Prefrail and Robust → Robust → Robust → Prefrail. Unfortunately, our sample size was insufficient to model these more granular changes. Similarly, we were not able to identify longer-term changes due to high amounts of death and attrition. Larger cohorts will be needed to assess whether claims data can be used to study more granular frailty paths and longer-term changes in frailty.

Our work should be interpreted in consideration of additional limitations. First, there was a large amount of missingness in the gold standard frailty measure. We attempted to account for this using hot-deck imputation, which has been shown to perform similarly to multiple imputation in the NHATS cohort (15). Second, we opted to include older adults in both the 2011 and 2015 NHATS cohorts to maximize sample size. This prevented us from incorporating the NHATS survey sampling weights to draw inference to a nationally representative sample of older Medicare beneficiaries. Finally, we only evaluated the Faurot frailty index and did not consider other claims-based frailty indices, which have been shown to perform similarly to the Faurot model at a single point in time (21). Future work should evaluate whether other claims-based frailty indices can better characterize clinically meaningful changes in frailty, including improvements in frailty.

Despite these challenges, we found that over a 3-year period, older adults who experienced worsening in the frailty phenotype also experienced worsening in the claims-based Faurot frailty index. Our results suggest that claims data may be used to identify clinically meaningful worsening in frailty. More work is needed to refine claims-based frailty scores to identify longitudinal improvements in frailty. In addition, future research should explore the use of claims-based frailty measures in specific disease settings, particularly to identify individuals at risk of frailty and to identify exposures associated with frailty progression.

## Supplementary Material

glae174_suppl_Supplementary_Material
