# Supplementary material for: Longitudinal Changes in a Claims-Based Frailty Proxy Measure Compared to Concurrent Changes in the Fried Frailty Phenotype
Source: J Gerontol A Biol Sci Med Sci. 2024 Jul 12;79(9):glae174. doi: 10.1093/gerona/glae174 (PMC11333825; doi:10.1093/gerona/glae174)

**Longitudinal changes in a claims-based frailty proxy measure compared to concurrent changes in the Fried frailty phenotype**

**Supplemental data file**

**Table of contents**

eMethods Additional details on development and validation of the Faurot frailty index

eFigure 1 Study schematic describing sensitivity analysis including death as its own health state

eFigure 2 Study consort diagram

eFigure 3 Results from sensitivity analysis treating death as its own health state. For individuals who died during follow-up, claims-based frailty was calculated using claims during the 240 days prior to the date of death

**eMethods**

**Additional details on development and validation of the Faurot frailty index**

The Faurot frailty index is a validated claims-based frailty proxy. The original model was developed using data linkage between the 2006 Medicare Current Beneficiary Survey (MCBS) and Medicare fee-for-service claims and enrollment data. ^1^ The development cohort included all older adults who participated in the 2006 MCBS who were living in the community, were 65 years of age and older, had continuous enrollment in Medicare fee-for-service for 8 months prior to the survey, and who were not missing data on functional status. Functional status was defined based on needing help with at least one of six activities of daily living (ADLs) using a modified Katz Index. Fifty-seven candidate predictors of functional dependence and their respective code lists were determined based on clinical expertise. Types of codes included International Classification of Diseases, Ninth Revision, Clinical Modification (ICD-9-CM), Current Procedural Terminology (CPT), and healthcare Common Procedure Coding System (HCPCS). The candidate predictors were identified using the 8 months of claims prior to the MCBS interview. Candidate predictors could be inversely associated with functional dependence (e.g., cancer screening). A multivariable logistic regression model with backwards elimination was used to determine the final set of frailty predictors. Bootstrapping with 1000 samples was used for internal validation; only predictors that were statistically significant (p≤0.05) in at least half of the bootstrapped samples were retained in the final model. The model was further validated by assessing associations between the claims-based predicted probability of frailty and five-year mortality.

The model was externally validated using data linkage between the Atherosclerosis Risk in Communities (ARIC) study and Medicare fee-for-service claims. ^2^ Phenotypic frailty was assessed using ARIC Visit 5 data based on the Fried frailty phenotype. The five components of the Fried frailty phenotype are weight loss, low physical activity, slow walking speed, exhaustion, and low grip strength. ^3^ Individuals are classified as frail if they meet at least 3 of the five components. The external validation found that the Faurot frailty index had good discrimination for the Fried frailty phenotype (C-statistic 0.71). Older adults with a high claims-based predicted probability of frailty (≥0.20) were more likely to experience falls, difficulties in physical abilities, and mortality.

The model was updated in 2023 for the ICD-10-CM era. ^4^ The code lists for the frailty predictors of the Faurot frailty index identified using ICD-9-CM codes were translated to the ICD-10-CM era using Centers for Medicare & Medicaid Services (CMS) General Equivalence Mappings (GEMs) and were manually reviewed for accuracy and consistency. An interrupted time series analysis of Medicare fee-for-service claims data was used to confirm that the distribution of the claims-based predicted probability of frailty was consistent across the transition. The updated model was also validated as a predictor of one-year outcomes that are relevant to a frail population, including skilled nursing facility admissions, hospitalizations, and one-year mortality. The updated model was also re-validated as a predictor of the concurrent Fried frailty phenotype in the Round 5 (2015) National Health and Aging Trends Study (NHATS) cohort with linkage to Medicare fee-for-service claims and enrollment data. ^5^ In the validation study, the Faurot frailty index had strong calibration and discrimination for the Fried frailty phenotype (C-statistic for 8-month frailty ascertainment window: 0.75).

**References**

1. Faurot KR, Jonsson Funk M, Pate V, et al. Using claims data to predict dependency in activities of daily living as a proxy for frailty. *Pharmacoepidemiol Drug Saf* 2015; 24: 59-66. 20141021. DOI: 10.1002/pds.3719.

2. Cuthbertson CC, Kucharska-Newton A, Faurot KR, et al. Controlling for Frailty in Pharmacoepidemiologic Studies of Older Adults: Validation of an Existing Medicare Claims-based Algorithm. *Epidemiology* 2018; 29: 556-561. DOI: 10.1097/EDE.0000000000000833.

3. Fried LP, Tangen CM, Walston J, et al. Frailty in older adults: evidence for a phenotype. *J Gerontol A Biol Sci Med Sci* 2001; 56: M146-156. DOI: 10.1093/gerona/56.3.m146.

4. Duchesneau ED, Shmuel S, Faurot KR, et al. Translation of a Claims-Based Frailty Index From the International Classification of Diseases, Ninth Revision, Clinical Modification to the Tenth Revision. Am J Epidemiol. Nov 10 2023;192(12):2085-2093. doi:10.1093/aje/kwad151

5. Duchesneau ED, Sturmer T, Kim DH, et al. Performance of a Claims-Based Frailty Proxy Using Varying Frailty Ascertainment Lookback Windows. Med Care. May 1 2024;62(5):305-313. doi:10.1097/MLR.0000000000001994

**eFigure 1. Study schematic describing sensitivity analysis including death as its own health state**

**
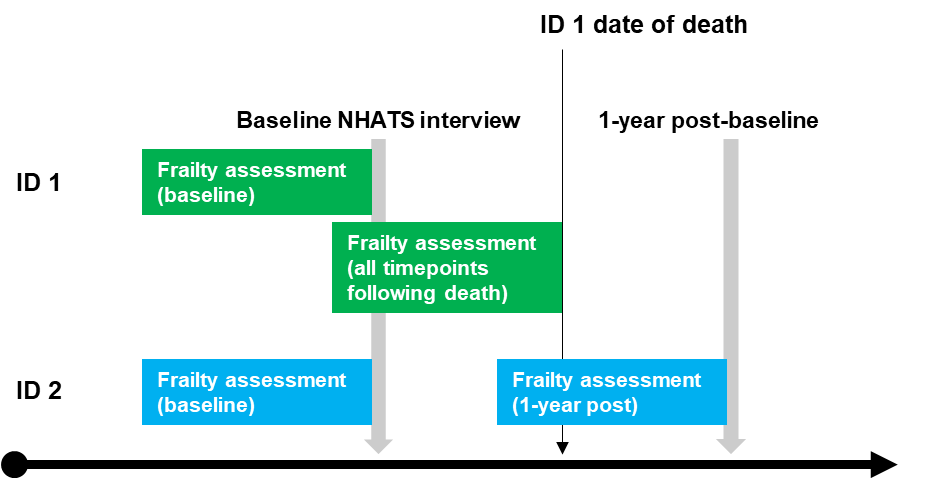
**

This figure depicts the assessment for claims-based frailty for individuals who died during follow-up in the sensitivity analysis including death as its own health state. The claims-based frailty assessment windows for ID 1 are depicted in **green** and the windows for ID 2 are depicted in **blue**. ID 1 died between the baseline NHATS interview and the 1-year post-baseline assessment. Their claims-based frailty score at the time of death is calculated using claims during the 240 days prior to and including their date of death. This claims-based frailty score is carried forward for all years of follow-up from the time of death onwards (i.e., this value would be included when calculating mean claims-based frailty in years 1, 2, and 3 post-baseline). Alternatively, ID 2 remains alive, and their frailty is assessed using claims on the date of their annual post-baseline NHATS surveys (see Figure 1 in main text).

**eFigure 2. Study consort diagram**


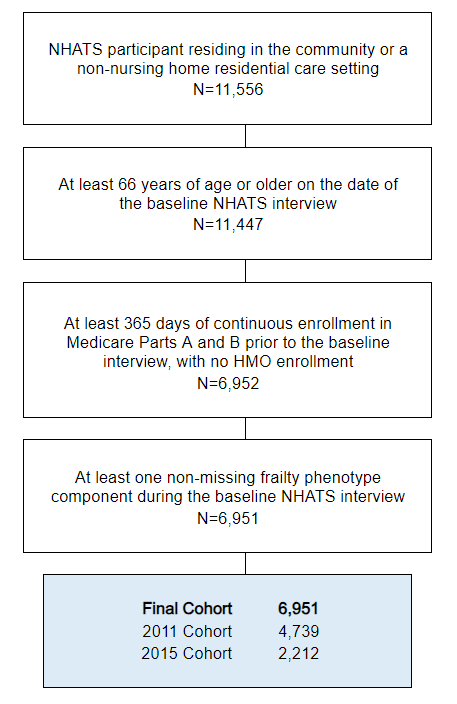


Abbreviations: HMO=health maintenance organization; NHATS=National Health and Aging Trends Study.

**eFigure 3. Results from sensitivity analysis treating death as its own health state. For individuals who died during follow-up, claims-based frailty was calculated using claims during the 240 days prior to the date of death**

A. Robust


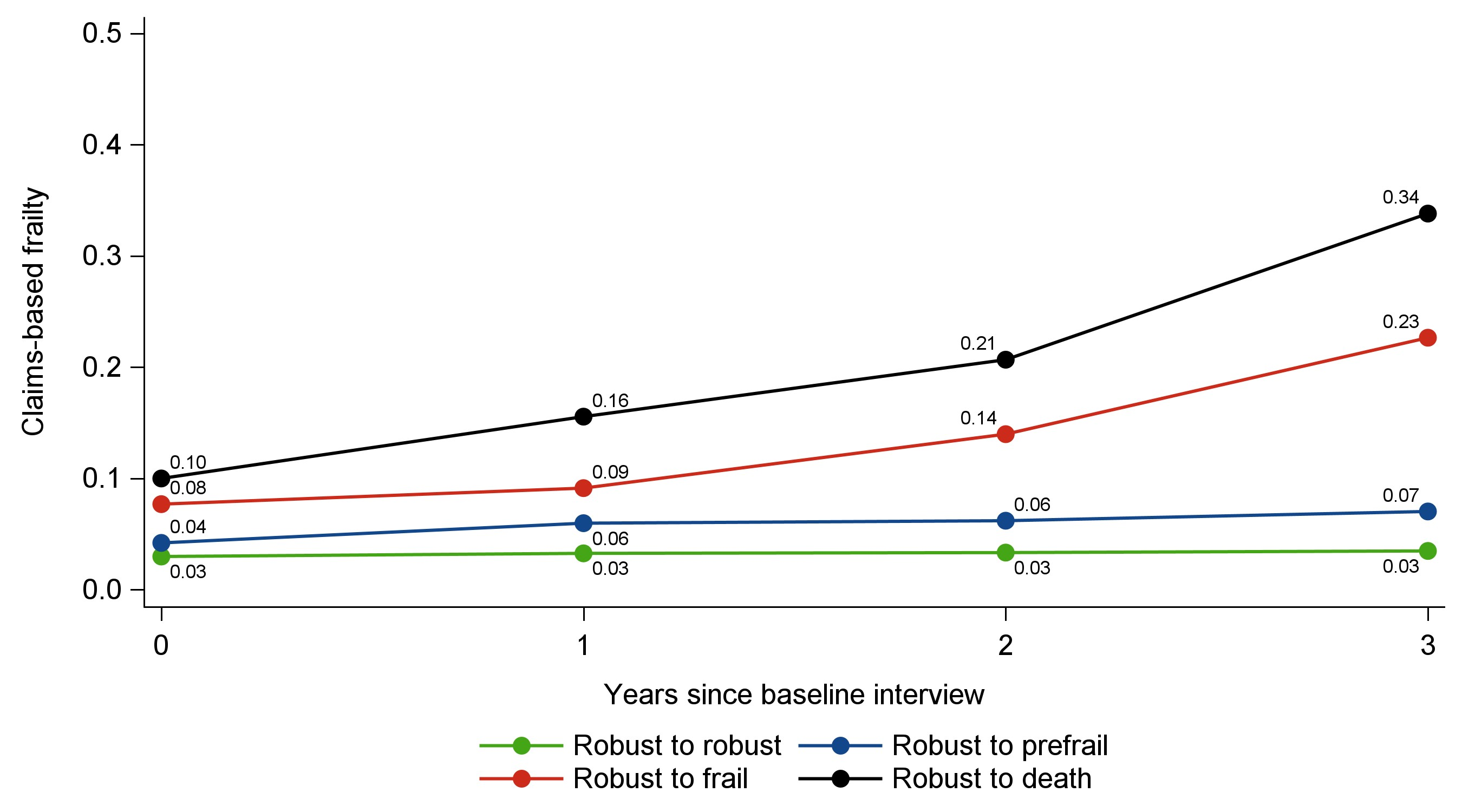


B. Prefrail


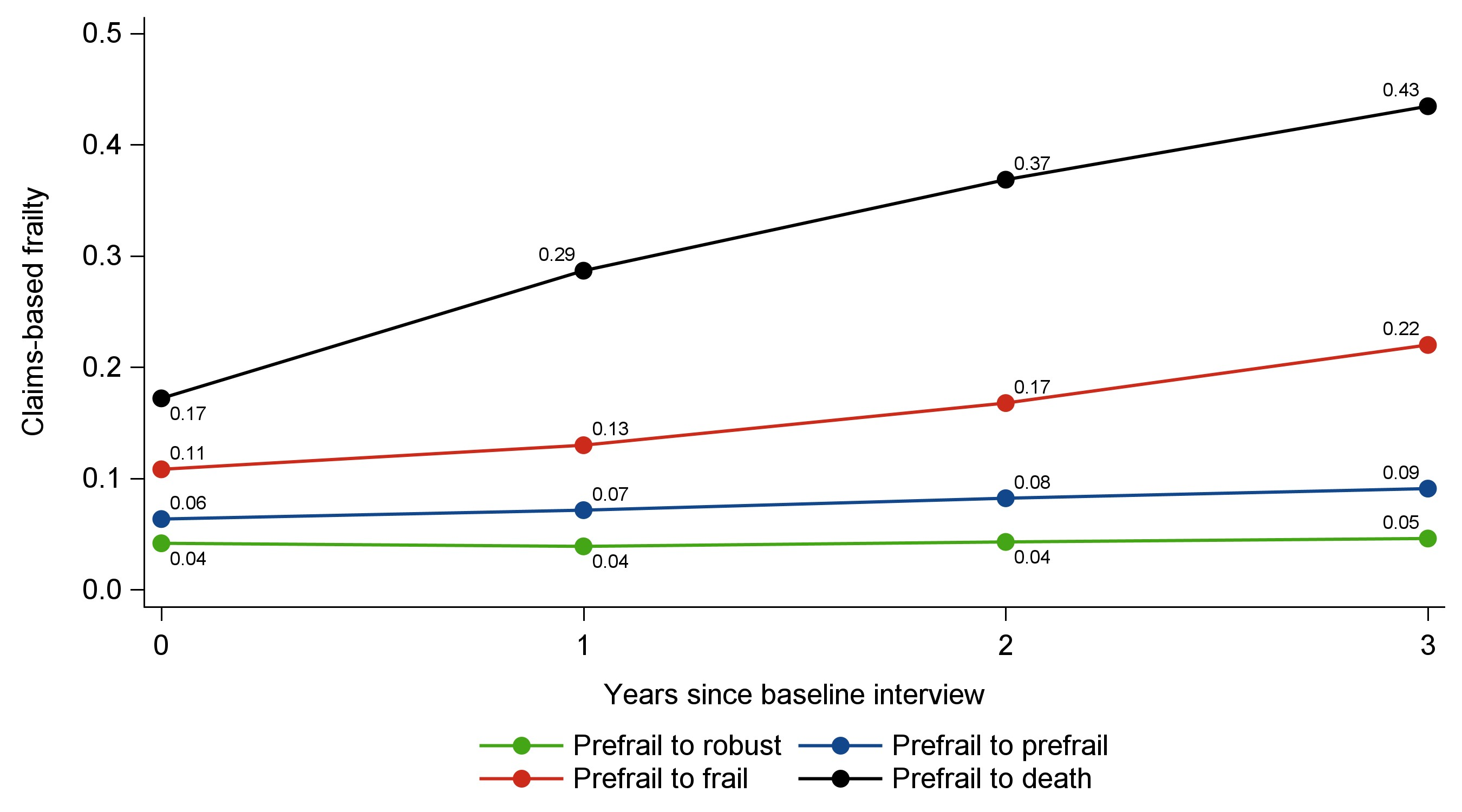


C. Frail


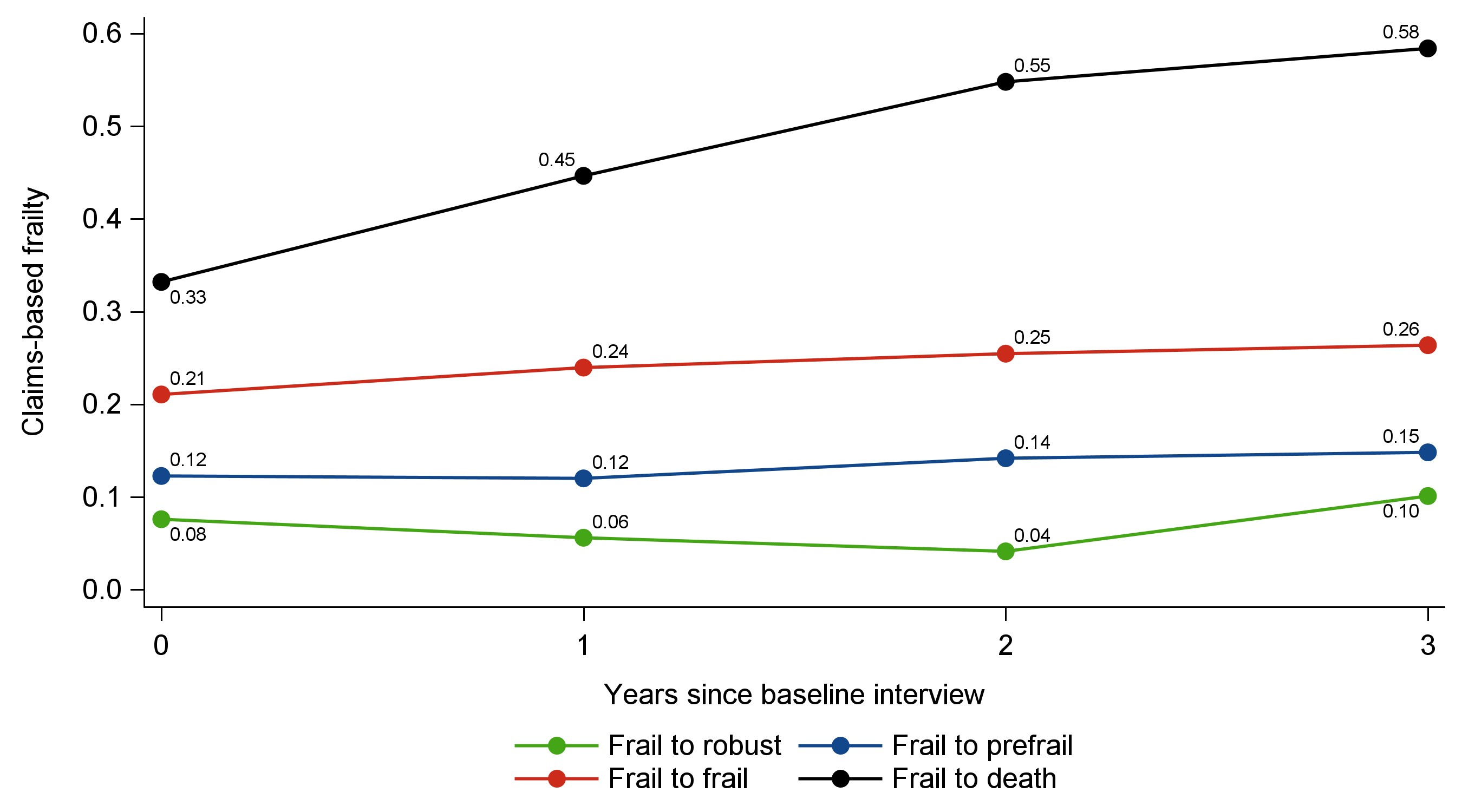

Supplement: glae174_suppl_Supplementary_Material [file glae174_suppl_supplementary_material.docx]
